# (*E*)-*N*′-[1-(Thio­phen-2-yl)ethyl­idene]isonicotinohydrazide

**DOI:** 10.1107/S1600536812039426

**Published:** 2012-09-22

**Authors:** C. S. Dileep, M. M. M Abdoh, M. P. Chakravarthy, K. N. Mohana, M. A. Sridhar

**Affiliations:** a Department of Studies in Physics, Manasagangotri, University of Mysore, Mysore 570 006, India; b Department of Physics, Faculty of Science, An Najah National University, Nablus, West Bank, Palestinian Territories; c Department of Studies in Chemistry, Manasagangotri, University of Mysore, Mysore 570 006, India

## Abstract

In the title compound, C_12_H_11_N_3_OS, the dihedral angle between the pyridine and thio­phene rings is 46.70 (9)° and the C—N—N—C torsion angle is 178.61 (15)°. In the crystal, inversion dimers linked by pairs of N—H⋯O hydrogen bonds generate *R*_2_^2^(8) loops.

## Related literature

For a related structure, see: Lu *et al.* (1996 ▶). For graph-set nomenclature of hydrogen bonds, see: Bernstein *et al.* (1995 ▶).
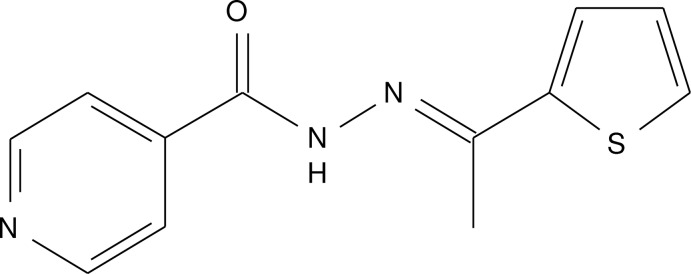


## Experimental

### 

#### Crystal data

C_12_H_11_N_3_OS
*M* *_r_* = 245.30
Triclinic, 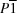

*a* = 3.9466 (1) Å
*b* = 10.5956 (4) Å
*c* = 14.3647 (6) Å
α = 74.656 (2)°
β = 82.595 (2)°
γ = 79.426 (2)°
*V* = 567.39 (4) Å^3^
*Z* = 2
Mo *K*α radiation
μ = 0.27 mm^−1^
*T* = 293 K
0.30 × 0.28 × 0.25 mm

#### Data collection

Bruker Kappa APEXII CCD diffractometer
Absorption correction: multi-scan (*SADABS*; Bruker, 2004 ▶) *T*_min_ = 0.853, *T*_max_ = 0.935
10651 measured reflections
2456 independent reflections
2162 reflections with *I* > 2σ(*I*)
*R*_int_ = 0.022

#### Refinement

*R*[*F* ^2^ > 2σ(*F* ^2^)] = 0.035
*wR*(*F* ^2^) = 0.104
*S* = 1.05
2456 reflections
159 parameters
1 restraint
H atoms treated by a mixture of independent and constrained refinement
Δρ_max_ = 0.26 e Å^−3^
Δρ_min_ = −0.20 e Å^−3^

### 

Data collection: *APEX2* (Bruker, 2004 ▶); cell refinement: *SAINT* (Bruker, 2004 ▶); data reduction: *SAINT*; program(s) used to solve structure: *SIR92* (Altomare *et al.*, 1993 ▶); program(s) used to refine structure: *SHELXL97* (Sheldrick, 2008 ▶); molecular graphics: *SHELXTL* (Sheldrick, 2008 ▶); software used to prepare material for publication: *PLATON* (Spek, 2009 ▶).

## Supplementary Material

Crystal structure: contains datablock(s) global, I. DOI: 10.1107/S1600536812039426/hb6959sup1.cif

Structure factors: contains datablock(s) I. DOI: 10.1107/S1600536812039426/hb6959Isup2.hkl

Additional supplementary materials:  crystallographic information; 3D view; checkCIF report

## Figures and Tables

**Table 1:** Hydrogen-bond geometry (Å, °)

*D*—H⋯*A*	*D*—H	H⋯*A*	*D*⋯*A*	*D*—H⋯*A*
N2—H2*A*⋯O1^i^	0.89 (1)	2.08 (1)	2.9561 (17)	170 (2)

Symmetry code: (i) 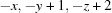.

**Fig. 1.**
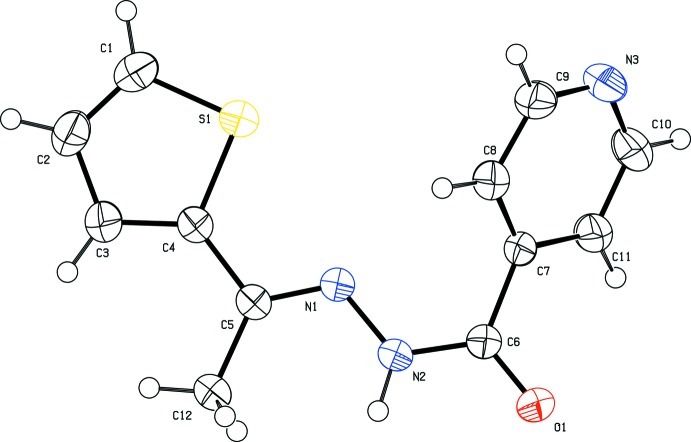
View of the title compound with displacement ellipsoids for non-H atoms drawn at the 50% probability level.

**Fig. 2.**
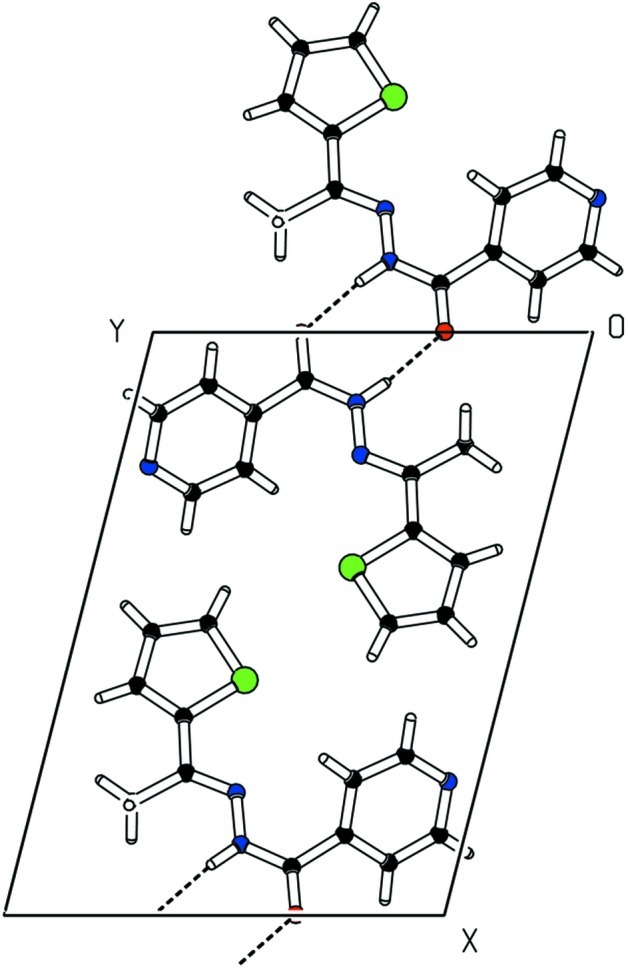
The unit-cell packing diagram.

## supplementary crystallographic information

### Comment

The geometry of C6 atom is distorted trigonal planar geometry as indicated by bond angles O1—C6—N2 = 120.77 (15) Å, (O1—C6—C7) = 119.27 (14) Å, (N2—C6—C7) = 119.96 (13) Å. The bond length of C6 and C7 = 1.496 (2) Å, which is comparable with an equivalent bond length of 1.506 (3) Å for a compound reported earlier (Lu *et al.*, 1996). The crystal structure exhibits intermolecular hydrogen bonds of the type N—H···O with symmetry codes -*x*, 1 - *y*, 2 - *z*.

### Experimental

1.37 g (10 mmol) of isoniazide was dissolved by the addition of 15 ml of ethanol in a round bottomed flask. To this 1.1 ml (10 mmol) of 2-Acetyl-thiophene dissolved in 15 ml of ethanol was added. This solution was refluxed for 5 h with stirring and then the solution was concentrated using rotor vaporizer and dried in vacuo and the product obtained was collected. The purity of the compound was confirmed by the TLC. Colourless blocks were recrystallised from methanol solution.

### Crystal data

**Table:**

C_12_H_11_N_3_OS	*Z* = 2
*M**_r_* = 245.30	*F*(000) = 256
Triclinic, *P*1	*D*_x_ = 1.436 Mg m^−^^3^
*a* = 3.9466 (1) Å	Mo *K*α radiation, λ = 0.71073 Å
*b* = 10.5956 (4) Å	Cell parameters from 5900 reflections
*c* = 14.3647 (6) Å	θ = 2.2–31.0°
α = 74.656 (2)°	µ = 0.27 mm^−^^1^
β = 82.595 (2)°	*T* = 293 K
γ = 79.426 (2)°	Block, colourless
*V* = 567.39 (4) Å^3^	0.30 × 0.28 × 0.25 mm

### Data collection

**Table:**

Bruker Kappa APEXII CCD diffractometer	2456 independent reflections
Radiation source: fine-focus sealed tube	2162 reflections with *I* > 2σ(*I*)
Graphite monochromator	*R*_int_ = 0.022
ω and φ scans	θ_max_ = 27.0°, θ_min_ = 2.2°
Absorption correction: multi-scan (*SADABS*; Bruker, 2004)	*h* = −5→4
*T*_min_ = 0.853, *T*_max_ = 0.935	*k* = −13→13
10651 measured reflections	*l* = −18→18

### Refinement

**Table:**

Refinement on *F*^2^	Primary atom site location: structure-invariant direct methods
Least-squares matrix: full	Secondary atom site location: difference Fourier map
*R*[*F*^2^ > 2σ(*F*^2^)] = 0.035	Hydrogen site location: inferred from neighbouring sites
*wR*(*F*^2^) = 0.104	H atoms treated by a mixture of independent and constrained refinement
*S* = 1.05	*w* = 1/[σ^2^(*F*_o_^2^) + (0.0524*P*)^2^ + 0.2115*P*] where *P* = (*F*_o_^2^ + 2*F*_c_^2^)/3
2456 reflections	(Δ/σ)_max_ < 0.001
159 parameters	Δρ_max_ = 0.26 e Å^−^^3^
1 restraint	Δρ_min_ = −0.20 e Å^−^^3^

### Special details

**Table:**

Geometry. All e.s.d.'s (except the e.s.d. in the dihedral angle between two l.s. planes) are estimated using the full covariance matrix. The cell e.s.d.'s are taken into account individually in the estimation of e.s.d.'s in distances, angles and torsion angles; correlations between e.s.d.'s in cell parameters are only used when they are defined by crystal symmetry. An approximate (isotropic) treatment of cell e.s.d.'s is used for estimating e.s.d.'s involving l.s. planes.
Refinement. Refinement of *F*^2^ against ALL reflections. The weighted *R*-factor *wR* and goodness of fit *S* are based on *F*^2^, conventional *R*-factors *R* are based on *F*, with *F* set to zero for negative *F*^2^. The threshold expression of *F*^2^ > σ(*F*^2^) is used only for calculating *R*-factors(gt) *etc*. and is not relevant to the choice of reflections for refinement. *R*-factors based on *F*^2^ are statistically about twice as large as those based on *F*, and *R*-factors based on ALL data will be even larger.

### Fractional atomic coordinates and isotropic or equivalent isotropic displacement parameters (Å^2^)

**Table:**

	*x*	*y*	*z*	*U*_iso_*/*U*_eq_	
C1	0.9693 (5)	0.70554 (18)	0.50027 (13)	0.0472 (4)	
H1	1.0867	0.6862	0.4441	0.057*	
C2	0.8919 (5)	0.82803 (19)	0.51454 (13)	0.0499 (4)	
H2	0.9499	0.9031	0.4691	0.060*	
C3	0.7129 (5)	0.83102 (17)	0.60598 (12)	0.0446 (4)	
H3	0.6397	0.9080	0.6273	0.053*	
C4	0.6596 (4)	0.70697 (14)	0.65967 (11)	0.0323 (3)	
C5	0.4812 (4)	0.66887 (14)	0.75589 (10)	0.0310 (3)	
C6	0.2859 (4)	0.37449 (15)	0.91748 (11)	0.0351 (3)	
C7	0.4383 (4)	0.27417 (13)	0.86131 (10)	0.0310 (3)	
C8	0.3769 (4)	0.28667 (15)	0.76656 (11)	0.0387 (4)	
H8	0.2533	0.3641	0.7313	0.046*	
C9	0.5030 (5)	0.18159 (17)	0.72543 (12)	0.0443 (4)	
H9	0.4566	0.1904	0.6620	0.053*	
C10	0.7437 (5)	0.05892 (16)	0.86100 (14)	0.0483 (4)	
H10	0.8728	−0.0189	0.8939	0.058*	
C11	0.6241 (4)	0.15680 (15)	0.90973 (12)	0.0400 (4)	
H11	0.6679	0.1439	0.9739	0.048*	
C12	0.3178 (5)	0.77498 (15)	0.80545 (12)	0.0415 (4)	
H12A	0.0709	0.7799	0.8113	0.062*	
H12B	0.3787	0.8585	0.7682	0.062*	
H12C	0.3985	0.7549	0.8688	0.062*	
N1	0.4799 (3)	0.54402 (12)	0.78951 (9)	0.0342 (3)	
N2	0.3047 (4)	0.50324 (12)	0.87852 (9)	0.0380 (3)	
N3	0.6861 (4)	0.06878 (14)	0.77044 (11)	0.0473 (4)	
O1	0.1424 (4)	0.33773 (12)	0.99877 (9)	0.0522 (3)	
S1	0.82822 (12)	0.58973 (4)	0.59655 (3)	0.04475 (15)	
H2A	0.188 (5)	0.5583 (17)	0.9127 (13)	0.055 (6)*	

### Atomic displacement parameters (Å^2^)

**Table:**

	*U*^11^	*U*^22^	*U*^33^	*U*^12^	*U*^13^	*U*^23^
C1	0.0532 (10)	0.0516 (10)	0.0356 (8)	−0.0122 (8)	0.0064 (7)	−0.0107 (7)
C2	0.0639 (12)	0.0450 (10)	0.0392 (9)	−0.0203 (8)	0.0043 (8)	−0.0038 (7)
C3	0.0596 (11)	0.0350 (8)	0.0399 (9)	−0.0144 (7)	0.0031 (7)	−0.0094 (7)
C4	0.0362 (8)	0.0286 (7)	0.0326 (7)	−0.0052 (6)	−0.0022 (6)	−0.0087 (6)
C5	0.0339 (7)	0.0276 (7)	0.0319 (7)	−0.0042 (5)	−0.0020 (6)	−0.0091 (6)
C6	0.0424 (8)	0.0286 (7)	0.0313 (7)	−0.0031 (6)	0.0050 (6)	−0.0074 (6)
C7	0.0346 (7)	0.0259 (7)	0.0311 (7)	−0.0068 (5)	0.0054 (6)	−0.0071 (5)
C8	0.0479 (9)	0.0322 (8)	0.0343 (8)	−0.0059 (6)	−0.0018 (7)	−0.0064 (6)
C9	0.0576 (10)	0.0439 (9)	0.0358 (8)	−0.0150 (8)	0.0013 (7)	−0.0154 (7)
C10	0.0560 (11)	0.0309 (8)	0.0542 (10)	0.0054 (7)	−0.0039 (8)	−0.0122 (7)
C11	0.0502 (9)	0.0327 (8)	0.0344 (8)	−0.0008 (7)	−0.0036 (7)	−0.0075 (6)
C12	0.0523 (10)	0.0300 (7)	0.0415 (8)	−0.0063 (7)	0.0073 (7)	−0.0132 (6)
N1	0.0429 (7)	0.0275 (6)	0.0304 (6)	−0.0052 (5)	0.0051 (5)	−0.0082 (5)
N2	0.0510 (8)	0.0263 (6)	0.0332 (7)	−0.0032 (5)	0.0099 (6)	−0.0099 (5)
N3	0.0562 (9)	0.0377 (7)	0.0514 (9)	−0.0066 (6)	0.0049 (7)	−0.0217 (6)
O1	0.0758 (9)	0.0345 (6)	0.0369 (6)	−0.0040 (6)	0.0215 (6)	−0.0084 (5)
S1	0.0584 (3)	0.0344 (2)	0.0389 (2)	−0.00466 (18)	0.00777 (18)	−0.01196 (17)

### Geometric parameters (Å, º)

**Table:**

C1—C2	1.341 (3)	C7—C11	1.381 (2)
C1—S1	1.6977 (18)	C8—C9	1.380 (2)
C1—H1	0.9300	C8—H8	0.9300
C2—C3	1.414 (2)	C9—N3	1.328 (2)
C2—H2	0.9300	C9—H9	0.9300
C3—C4	1.375 (2)	C10—N3	1.323 (2)
C3—H3	0.9300	C10—C11	1.381 (2)
C4—C5	1.458 (2)	C10—H10	0.9300
C4—S1	1.7162 (15)	C11—H11	0.9300
C5—N1	1.2834 (19)	C12—H12A	0.9600
C5—C12	1.492 (2)	C12—H12B	0.9600
C6—O1	1.2270 (18)	C12—H12C	0.9600
C6—N2	1.3425 (19)	N1—N2	1.3745 (17)
C6—C7	1.4956 (19)	N2—H2A	0.888 (9)
C7—C8	1.381 (2)		
			
C2—C1—S1	112.29 (14)	C7—C8—H8	120.8
C2—C1—H1	123.9	N3—C9—C8	124.36 (16)
S1—C1—H1	123.9	N3—C9—H9	117.8
C1—C2—C3	112.91 (16)	C8—C9—H9	117.8
C1—C2—H2	123.5	N3—C10—C11	124.17 (16)
C3—C2—H2	123.5	N3—C10—H10	117.9
C4—C3—C2	112.15 (15)	C11—C10—H10	117.9
C4—C3—H3	123.9	C7—C11—C10	118.75 (15)
C2—C3—H3	123.9	C7—C11—H11	120.6
C3—C4—C5	128.92 (14)	C10—C11—H11	120.6
C3—C4—S1	110.69 (12)	C5—C12—H12A	109.5
C5—C4—S1	120.38 (11)	C5—C12—H12B	109.5
N1—C5—C4	115.18 (13)	H12A—C12—H12B	109.5
N1—C5—C12	126.20 (14)	C5—C12—H12C	109.5
C4—C5—C12	118.62 (13)	H12A—C12—H12C	109.5
O1—C6—N2	120.77 (14)	H12B—C12—H12C	109.5
O1—C6—C7	119.27 (13)	C5—N1—N2	117.49 (12)
N2—C6—C7	119.96 (13)	C6—N2—N1	120.90 (12)
C8—C7—C11	117.96 (13)	C6—N2—H2A	115.3 (14)
C8—C7—C6	123.76 (13)	N1—N2—H2A	123.7 (14)
C11—C7—C6	117.98 (13)	C10—N3—C9	116.26 (14)
C9—C8—C7	118.49 (15)	C1—S1—C4	91.96 (8)
C9—C8—H8	120.8		
			
S1—C1—C2—C3	0.1 (2)	C7—C8—C9—N3	−1.2 (3)
C1—C2—C3—C4	0.2 (3)	C8—C7—C11—C10	1.0 (2)
C2—C3—C4—C5	−178.72 (16)	C6—C7—C11—C10	175.01 (15)
C2—C3—C4—S1	−0.3 (2)	N3—C10—C11—C7	−1.4 (3)
C3—C4—C5—N1	−178.08 (16)	C4—C5—N1—N2	−177.77 (13)
S1—C4—C5—N1	3.7 (2)	C12—C5—N1—N2	1.8 (2)
C3—C4—C5—C12	2.3 (3)	O1—C6—N2—N1	176.75 (16)
S1—C4—C5—C12	−175.92 (12)	C7—C6—N2—N1	−3.7 (2)
O1—C6—C7—C8	130.37 (18)	C5—N1—N2—C6	178.61 (15)
N2—C6—C7—C8	−49.1 (2)	C11—C10—N3—C9	0.5 (3)
O1—C6—C7—C11	−43.3 (2)	C8—C9—N3—C10	0.8 (3)
N2—C6—C7—C11	137.23 (16)	C2—C1—S1—C4	−0.23 (16)
C11—C7—C8—C9	0.2 (2)	C3—C4—S1—C1	0.32 (14)
C6—C7—C8—C9	−173.46 (15)	C5—C4—S1—C1	178.87 (14)

### Hydrogen-bond geometry (Å, º)

**Table:**

*D*—H···*A*	*D*—H	H···*A*	*D*···*A*	*D*—H···*A*
N2—H2*A*···O1^i^	0.89 (1)	2.08 (1)	2.9561 (17)	170 (2)

Symmetry code: (i) −*x*, −*y*+1, −*z*+2.
